# Electron Microscopic, Genetic and Protein Expression Analyses of *Helicobacter acinonychis* Strains from a Bengal Tiger

**DOI:** 10.1371/journal.pone.0071220

**Published:** 2013-08-05

**Authors:** Nicole Tegtmeyer, Francisco Rivas Traverso, Manfred Rohde, Omar A. Oyarzabal, Norbert Lehn, Wulf Schneider-Brachert, Richard L. Ferrero, James G. Fox, Douglas E. Berg, Steffen Backert

**Affiliations:** 1 Institute of Medical Microbiology, Otto von Guericke University Magdeburg, Magdeburg, Germany; 2 Helmholtz Centre for Infection Research, Braunschweig, Germany; 3 Institute for Environmental Health, Inc., Seattle, Washington, United States of America; 4 Institute for Medical Microbiology and Hygiene, University of Regensburg, Regensburg, Germany; 5 Centre for Innate Immunity & Infectious Diseases, Monash Institute of Medical Research, Clayton, Australia; 6 Division of Comparative Medicine, Massachusetts Institute of Technology, Cambridge, Massachusetts, United States of America; 7 Department of Molecular Microbiology, Washington University School of Medicine, St. Louis, Missouri, United States of America; University of Osnabrueck, Germany

## Abstract

Colonization by *Helicobacter* species is commonly noted in many mammals. These infections often remain unrecognized, but can cause severe health complications or more subtle host immune perturbations. The aim of this study was to isolate and characterize putative novel *Helicobacter* spp. from Bengal tigers in Thailand. Morphological investigation (Gram-staining and electron microscopy) and genetic studies (16SrRNA, 23SrRNA, flagellin, urease and prophage gene analyses, RAPD DNA fingerprinting and restriction fragment polymorphisms) as well as Western blotting were used to characterize the isolated *Helicobacters*. Electron microscopy revealed spiral-shaped bacteria, which varied in length (2.5–6 µm) and contained up to four monopolar sheathed flagella. The 16SrRNA, 23SrRNA, sequencing and protein expression analyses identified novel *H. acinonychis* isolates closely related to *H. pylori*. These Asian isolates are genetically very similar to *H. acinonychis* strains of other big cats (cheetahs, lions, lion-tiger hybrid and other tigers) from North America and Europe, which is remarkable in the context of the great genetic diversity among worldwide *H. pylori* strains. We also found by immunoblotting that the Bengal tiger isolates express UreaseA/B, flagellin, BabA adhesin, neutrophil-activating protein NapA, HtrA protease, γ-glutamyl-transpeptidase GGT, Slt lytic transglycosylase and two DNA transfer relaxase orthologs that were known from *H. pylori*, but not the *cag* pathogenicity island, nor CagA, VacA, SabA, DupA or OipA proteins. These results give fresh insights into *H. acinonychis* genetics and the expression of potential pathogenicity-associated factors and their possible pathophysiological relevance in related gastric infections.

## Introduction

The genus *Helicobacter* comprises a heterogeneous group of Gram-negative bacteria that colonise different mammalian hosts, including domestic and wild animals, non-human primates and humans [1], [2]. Currently there are 33 validated *Helicobacter* species and several other described isolated candidates [2]. Best known is the human gastric pathogen, *Helicobacter pylori* [3], which is highly motile using a unipolar bundle of two to six sheathed flagella [4]. During co-evolution with humans, a multitude of pathogenicity-associated factors were developed to adapt and survive in the challenging gastric milieu. *Helicobacter pylori’*s hallmark enzyme is a potent multi-subunit urease complex, which is fundamental for neutralizing the acidic pH in the stomach [5]. Other bacterial factors such as the blood-group antigen binding protein BabA [6], sialic acid-binding adhesin SabA [7], outer inflammatory protein OipA [8], neutrophil activating protein NapA [9]–[11], lytic transglycosylase Slt [12], duodenal ulcer promoter protein A (DupA) [13]–[15] and γ-glutamyl transpeptidase (GGT) [16] contribute significantly to successful *H. pylori* pathogenesis. In addition, the secreted protease HtrA (high temperature requirement A) may disrupt epithelial cell barrier functions as it can cleave the host tumor suppressor and cell adhesion protein E-cadherin [17], [18]; and two potential relaxases, the VirD2 homologs Rlx1 and Rlx2, are involved in DNA transfer [19], [20]. The best studied virulence factors in *H. pylori*, however, are the vacuolating cytotoxin VacA and the effector protein CagA [21], [22]. Mature VacA is a multifunctional toxin implicated in perturbing endosomal trafficking, mitochondrial apoptosis and inhibition of T-cell proliferation [23], [24]. The *cagA* gene, located in the *cag* pathogenicity island (*cag*PAI), is a marker of a type IV secretion system, a molecular syringe-like structure (composed of VirB1 to VirB11, VirD4 and several other Cag proteins) through which CagA can be delivered into host target cells [25].

Established genetic polymorphisms in *cagA* and *vacA* genes affect *H. pylori* infection outcomes and also exhibit clear phylogeographical structural differences that reflect both ancient and recent human migrations and contacts [22], [26]. More generally, *H. pylori* is genetically highly diverse; independent isolates (from unrelated persons) are usually distinguishable from one another by DNA fingerprinting [27]; and strains typically differ from one another by some 2–5% in sequences of essential housekeeping genes and 5% or more in overall gene content [28], [29]. This stems from frequent point mutation, differences in restriction-modification systems, and recombination between divergent strains and species. *H. pylori* is transmitted preferentially within families and communities [30], and phylogenetically distinct sets of DNA sequences are found in strains from different parts of the world [26]–[29].

Genetic studies indicate that *H. pylori* co-migrated with humans from east Africa around 58,000 years ago, and its present worldwide genetic diversity reflects the isolation by distance that has shaped this bacterial species over time [26]. However, it is still not fully clear when exactly *H. pylori* became adapted to the human gastric niche. The most favoured idea is that *Helicobacter* species have been universally part of humans and our non-human primate ancestors’ microbiota since long before modern *Homo sapiens* appeared on Earth [31], [32]. Alternatively, a host jump theory [33] suggested acquisition of *H. pylori* infections in humans more recently, ca. 10,000 years ago, when the first agricultural societies started, as the result of frequent contacts with infected domesticated animals [34]. Besides scattered reports of natural *H. pylori* infection associated gastritis in domestic cats [2], [35]–[37], the main natural hosts for *H. pylori* seem to be humans and some non-human primates [38], [39]. The potential for species jumps between hosts is illustrated, however, by lab reports of human *H. pylori* strains that were adapted to mice, dogs, cats, piglets and mongolian gerbils [40]–[43]. In addition, a number of other gastric non-pylori *Helicobacter* spp. have been identified in various mammalian hosts in recent years, including *H. felis, H. acinonychis*, *H. salomonis, H. bizzozeronii*, *H. mustelae, H. suis* and *H. aurati* as well as some extragastric *Helicobacter* spp. such as *H. canis*, *H. pullorum*, *H. cinaedi*, *H. fennelliae*, *H. typhlonius*, *H. bilis*, *H. hepaticus* and *H. magdeburgensis* [1], [2], [38], [44]–[47].

Based on genome sequencing and other reports, the closest relative of *H. pylori* is *H. acinonychis,* which has been found in stomachs of big predator cats such as lions or cheetahs [31], [33], [44], [48]–[52]. Complete sequencing of *H. acinonychis* strain Sheeba and comparisons to European and African *H. pylori* strains exhibited similar core genes and also distinctively different features including an unusually high number of fragmented genes for VacA and outer membrane proteins (OMPs). A host jump from early humans to large felines, probably about 200,000 years ago, was proposed [31]. However, our knowledge of non-pylori *Helicobacter* spp. such as *H. acinonychis* is still very incomplete, as illustrated by a total of only two deposited 16S rRNA sequences (accession numbers AM260522.1 and AF057163.1) for this species. We are interested in identifying novel gastric and extra-gastric *Helicobacter* spp., and characterising their genetics, bacterial pathogenicity factors and gastric disease-associated processes to better understand mechanisms of bacterial adaptation to host environments and associated health and disease [47]. Here we report on the isolation and detailed molecular characterisation of novel *H. acinonychis* strains from a Bengal tiger (*Panthera tigris tigris*).

## Results

### Isolation and Visualisation of *Helicobacter* Strains from a Bengal Tiger

**Figure 1:**
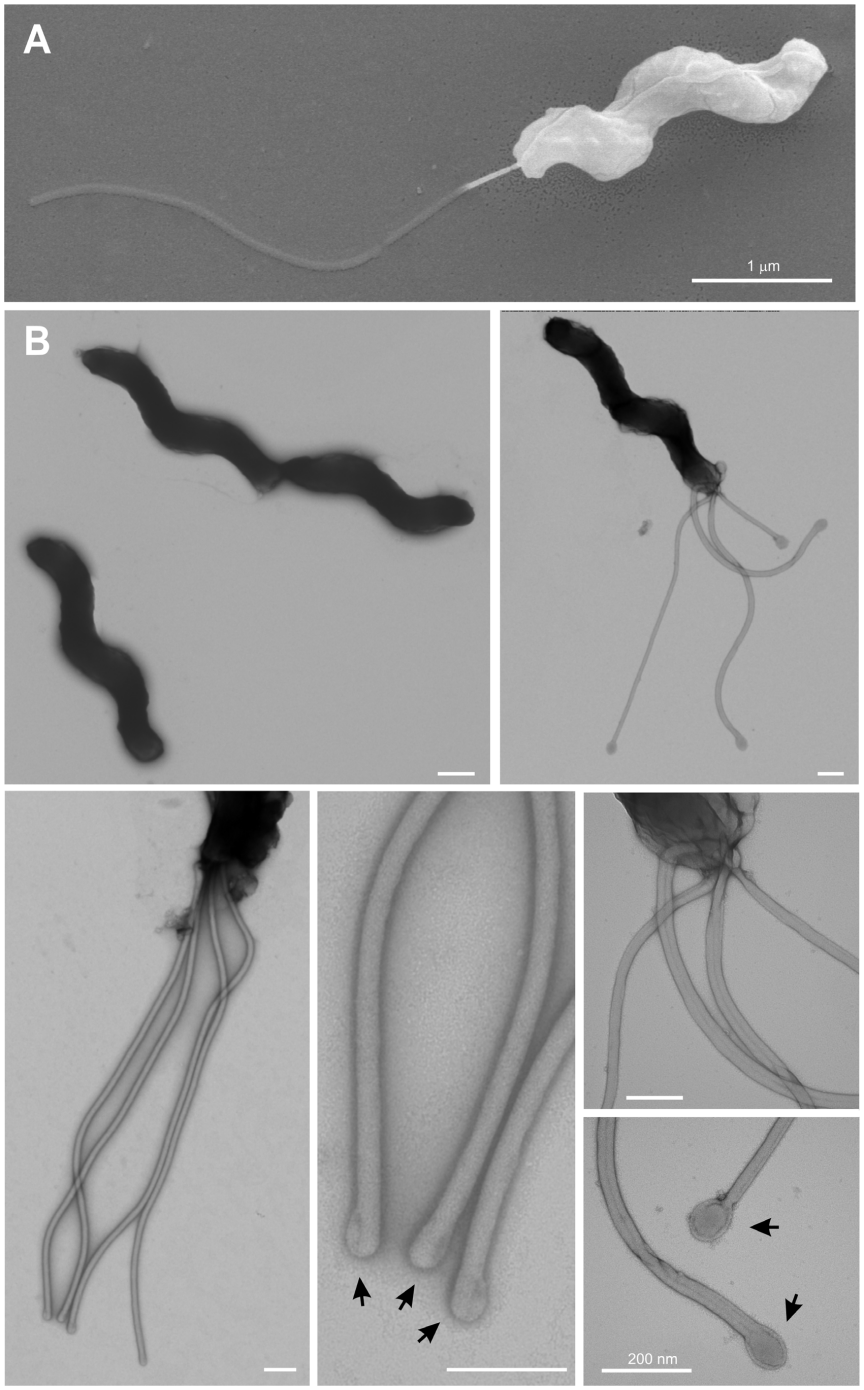
Morphological analyses of novel *Helicobacter* isolates from a Bengal tiger by two electron microscopic methods. Panel A: Scanning electron microscopy (SEM) revealed flagellated, spiral-shaped bacteria which were about 0.25–0.45 µm in diameter and varied in length from about 2.5–6 µm. Panel B: Negative contrast electron microscopy of the same *Helicobacter* samples. The majority of bacteria contained 1–4 monopolar flagella, while some bacteria were not flagellated as shown. Enlarged sections show the flagella and specific bulb-like structures at their tips. Representative pictures are shown from two preparations. The bars correspond to 1 µm (panel A) or 200 nm (panel B).

Five single *Helicobacter*-like colonies (called SB-1 to SB-5) from diarrheic fecal samples of a captive Bengal tiger from Thailand were grown under microaerobic growth conditions using either Columbia agar plates with 5% sheep blood or GC agar plates with 10% horse serum. Gram-staining indicated that these bacteria were Gram-negative. Scanning electron microscopic investigation revealed spiral-shaped *Helicobacter*-like organisms (Fig. 1A), about 0.25–0.45 µm in diameter and 2.5–6 µm in length. The majority of these bacteria contained 1–4 monopolar flagella with lengths of about 1.5–4.5 µm, although some were non-flagellated (Fig. S1). This suggests that some bacteria may have lost their flagella during sample processing. Negative stained samples revealed similar results (Fig. 1B). The visualized flagella were commonly sheathed and about 43–48 nm in diameter, although non-sheathed flagella (15–17 nm in diameter) were also observed. In addition, we often found that the flagella ends were thickened and had a bulb-like shape (see enlargement panels in Fig. 1B, arrows).

### 16S rRNA, 23S rRNA and RAPD Fingerprinting Analysis of *Helicobacter* Genomic DNA

**Figure 2:**
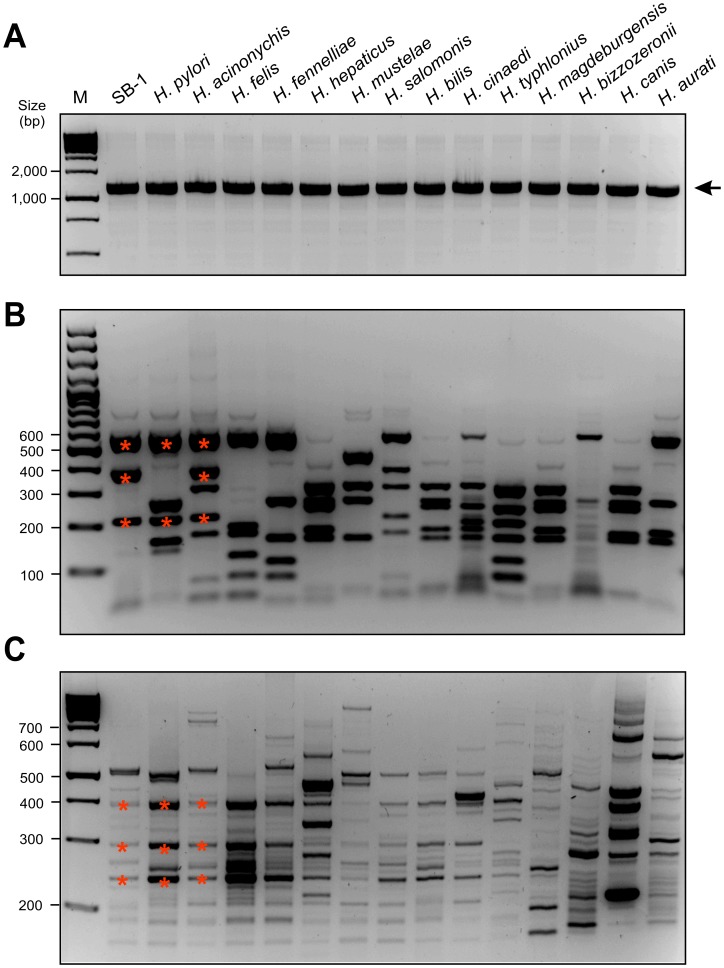
Analysis of 16S rRNA by PCR, RFLP and RAPD fingerprinting of different *Helicobacter* species. Panel A: DNA isolated from various *Helicobacter* species including the Bengal tiger isolate was subjected to 16SrRNA PCR. A conserved 1.5 kb DNA fragment of the genus *Helicobacter* was amplified [52]. Panel B: To confirm their specificity, all PCR products were then investigated by RFLP (restriction fragment length polymorphism) using the restriction endonuclease *Alu*I. The *Alu*I pattern of the tiger isolate gave rise to 3 major bands (asterisks) which were unique to all other *Helicobacters*. Some similar bands were only found in the RFLP pattern of *H. pylori* and *H. acinonychis* (asterisks), indicating their close genetic relatedness. Panel C: RAPD fingerprinting of the *Helicobacter* isolates using primer D9355 was performed as described previously [27]. A typical RAPD fingerprinting profile is shown and revealed the relatedness between *H. pylori*, *H. acinonychis* and Bengal tiger isolate. Asterisks indicate three major bands which were identical among the latter three samples. M, DNA size marker.

**Figure 3:**
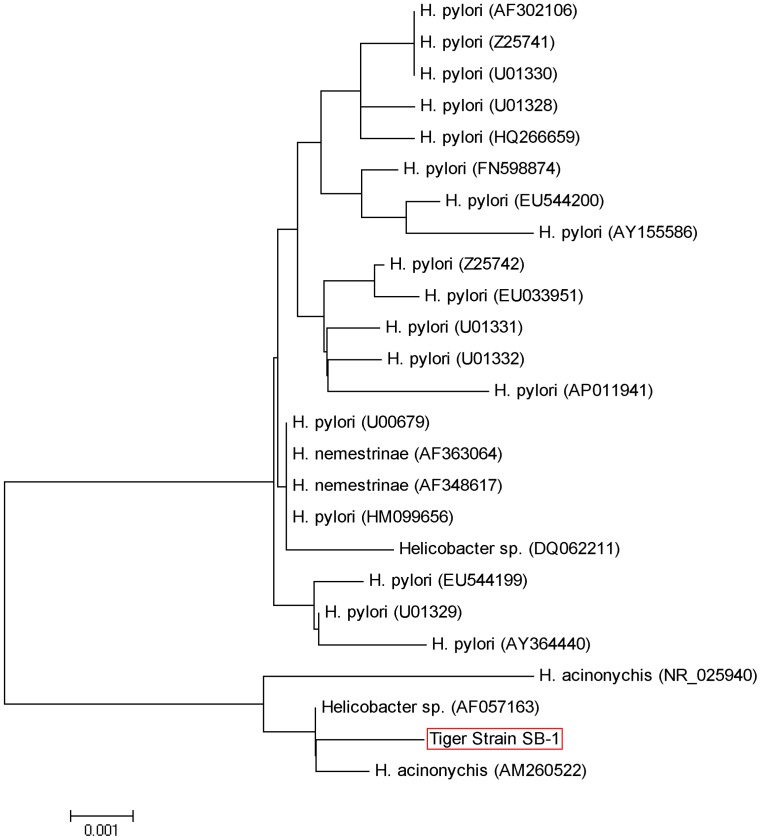
Phylogenetic tree of the 16S ribosomal RNA gene from the tiger strain SB-1 and the most closely related sequences from different *Helicobacter* species. The alignment was performed with BioEdit using gap penalties of 10 for gap opening and 5 for gap extension and a bootstrap value of 1,000. MEGA5 was used to infer DNA relatedness using the Neighbor-Joining method. The evolutionary distances were computed using the Maximum Composite Likelihood method and are in the units of the number of base substitutions per site. The optimal tree with the sum of branch length was equal to 0.0360 for 16S rRNA. *Helicobacter acinomychis* and *Helicobacter* spp. were used as outgroups. The phylogenetic tree shows that the 16SrRNA gene of our Bengal tiger strain (accession number JN251811.1) branched together with *H. acinonychis* from Sumatra tiger (AM260522), thus demonstrating close relatedness among them.

Next, we investigated the 16S rRNA of these isolates in direct comparison with various Helicobacter species including *H. pylori*, *H. acinonychis*, *H. felis*, *H. fennelliae*, *H. hepaticus*, *H. mustelae*, *H. salomonis*, *H. bilis*, *H. cinaedi*, *H. typhlonius*, *H. magdeburgensis*, *H. bizzozeroni*, *H. canis* and *H. aurati*. For this purpose, we amplified a 1.2 kb DNA fragment of the 16S rRNA region which is highly conserved within the genus Helicobacter [53]. All Helicobacter species revealed the expected PCR product, while Campylobacter jejuni or other controls did not (Fig. 2A and data not shown). To confirm the specificity of these fragments, all PCR products were then digested with the restriction endonuclease HhaI or AluI, which yield specific band patterns for known Helicobacter species [54]. The RFLP pattern was similar between various Helicobacter isolates (Fig. 2B and Fig. S2A–B), and SB-1 was most similar to those of *H. pylori* and *H. acinonychis*, which suggests that our Bengal tiger isolates are closely related to these species. This conclusion is in good agreement with the RAPD fingerprinting profiles using various primers (Fig. 2C and Fig. S2C–D). The 16S rRNA genes from our individual isolates were then sequenced (GenBank accession number JN251811.1). They belong phylogenetically to a specific 16S rRNA gene cluster, which includes isolates of the species H. acinonychis and H. pylori (Fig. 3A). Sequencing of a 23S rRNA gene fragment yielded a dendrogram which was also in full agreement with that generated by the analysis of the 16S rRNA gene (Fig. S3).

### Bengal Tiger *Helicobacter* Isolates Express a Functional Urease

**Figure 4:**
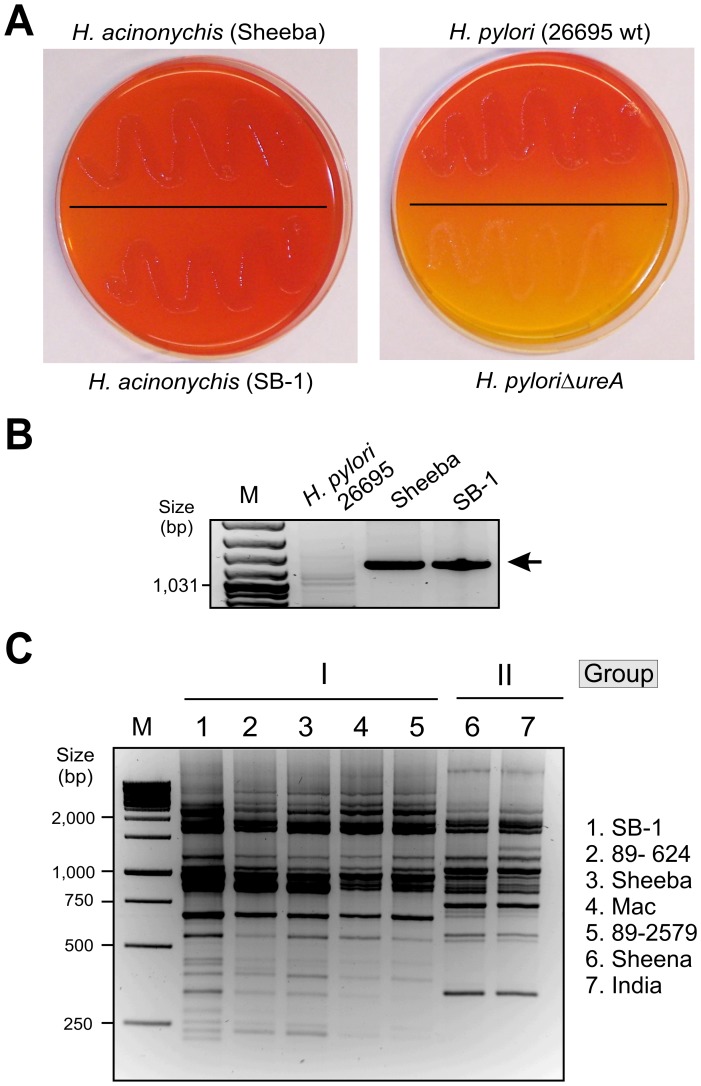
Urease expression and genetic relatedness of various *Helicobacter acinonychis* isolates from different big cats from the US, Europe and Asia. Panel A: Selection of bacteria producing functional urease on acidified agar supplemented with urea [55]. Left samples correspond to *H. acinonychis* strain Sheeba (top) and the Bengal tiger isolate SB-1 (bottom). The observed color change from orange to red indicated that bacterial colonies were producing functional urease and growing. Right samples are the *H. pylori* wild-type (wt) 26695 (top) and isogenic Δ*ureA* mutant (bottom). Color change did not occur in the Δ*ureA* mutant, indicating that functional urease was not being produced. Panel B: PCR of the prophage gene helicase (Hac1336, Supplemental Table S1) shows a specific 1.2 kb product for the Bengal tiger isolate SB-1 and other *H. acinonychis* strains, but not *H. pylori*. Panel C: RAPD fingerprinting of the indicated *H. acinonychis* isolates from tigers, cheetahs, lions and lion-tiger reveals the close relatedness between strains in two specific groups, called I and II, as indicated. The RAPD primer D1254 [27] has been used in this experiment. M, DNA size marker.

An active urease is considered essential for all *Helicobacters* that colonize the acidic mammalian stomach, whereas extra-gastric *Helicobacters* are generally without urease [1], [2]. To test the idea that our new *Helicobacters* are likely to be stomach colonizers despite having been isolated from feces, we next tested if our strains have a functional urease. For this purpose, bacteria were grown on selective acidified agar plates supplemented with urea, the substrate of *H. pylori* urease [55]. These experiments yielded functional urease enzymes allowing urea hydrolyzation to a high extent in all *H. acinonychis* strains including SB-1 to SB-5, similar to that of *H. pylori* control strain 26695, while retarded growth and no urea hydrolysis was seen in the 26695Δ*ureA* mutant as expected (Fig. 4A and data not shown). This confirms that the *Helicobacter* isolates exhibit strong urease activity, much like that of *H. pylori* and other *H. acinonychis* isolates, and thus, are likely of gastric origin although they also survived passage through the intestine.

### *Helicobacter* Isolates Contain Conserved Urease and Flagellin but not *vacA* or *cagA* Genes

Next we amplified and sequenced urease and flagellin genes (Table S1). Their sequences (deposited in the NCBI GenBank; accession numbers listed in Materials and Methods) are highly similar to those from *H. acinonychis* strain Sheeba (100% and 98% DNA identity, respectively), and less similar to corresponding *H. pylori* genes (94% and 90% identity, respectively, to those of strain 26695) (data not shown). As reported for various *H. acinonychis* strains from other big cats such as lions and cheetahs [33], analyses of PCR products using *vacA* gene specific primers indicated that *vacA* is fragmented, which implies that a functional vacuolating cytotoxin is not synthesized, an inference confirmed by immunoblotting (data not shown). Collectively, these data indicate that these tiger isolates belong to the novel *H. pylori*-derived species, *H. acinonychis*. The VacA results seem particularly remarkable since VacA is expressed in virtually all *H. pylori* strains and seems to contribute importantly to bacterial fitness during colonization [16]. Furthermore, we also failed to PCR amplify conserved fragments of *cag*A and other *cag*PAI genes such as *virB10* and *virB11* (Table S1), in accord with the lack of a *cag*PAI in the genome-sequenced *H. acinonychis* strain [31].

### Prophage Genes are Potential Genetic Markers for *Helicobacter acinonychis* Isolates

To further test our inference that these novel strains belong to the *H. acinonychis* group, we investigated the presence of several other genetic markers. Previous genome sequencing of the Sheeba isolate and subsequent microarray analyses had identified prophage genes [31], now known to be related to recently discovered plaque forming temperate phage from East Asia [56]–[58]. In general, certain prophages have been implicated in bacterial virulence [59] and it has been suggested that prophage genes of *H. acinonychis* might potentially affect host adaptation and specificity [31]. Forty one of the imported coding sequences (CDSs) in the Sheeba strain are actually present within two prophages, called prophage I and prophage II, but such prophages or remnants are present in only a very few *H. pylori* genomes [60]. A PCR assay developed for one of these prophage genes [prophage I ORF3, a helicase (Hac1336), Table S1], demonstrated this gene’s presence in SB-1 to SB-5, Sheeba and other *H. acinonychis* isolates, while its absence from several *H. pylori* in our collection (Fig. 4B). These results provide further evidence that SB-1 to SB-5 represent *H. acinonychis* strains.

### Genetic Comparison of *Helicobacter acinonychis* Isolates from Europe, US and Asia

To investigate genetic relatedness among different *H. acinonychis* isolates, we compared 16S rRNA genes and RAPD fingerprint typing patterns of our five isolates (SB-1 and SB-5) with those of two other *H. acinonychis* strains from a Sumatran tiger maintained in captivity in a German zoo [61]. RFLP of the 16S rRNA PCR products yielded identical fragment patterns (Fig. S4A–D). The nucleotide sequence of the 16S rRNA gene was also highly similar between isolates from Bengal and Sumatran tigers (accession number AF057163) (Fig. 3, bottom). Thus, we next scored RAPD fingerprint patterns, which are more effective than the focused analysis of individual genes at discriminating between related strains. The RAPD patterns were also almost identical between our five strains and those from the Bengal and Sumatran tigers (Fig. S5A–C), indicating that very closely related *H. acinonychis* strains have colonized different tiger subspecies from very different geographic locations.

Next, we compared the RAPD fingerprint patterns of our *H. acinonychis* strain SB-1 with those of strains from other big cats: namely three cheetahs from a US zoo, two lions, one lion-tiger hybrid and another tiger housed at a European circus (Table 1). The results show that the fingerprinting patterns of our isolates from the Bengal tiger are also very similar to those of the cheetah and lion isolates, which were categorised in group I [33], while are distinct from those of the lion-tiger hybrid and other tiger isolates, classified as group II *H. acinonychis* (Fig. 4C and Fig. S6A–C) [33].

**Table 1:** Origin and characteristics of *Helicobacter acinonychis* strains investigated in this study.

Strain	Infected animal^a^	Origin country	Sample origin	RAPD group	Reference
SB-1 to SB-5	Bengal tiger	Zoo, Thailand	Diarrheic sample	I	This study
90–624	Cheetah	Zoo, Columbus, Ohio, USA	Stomach biopsy	I	[44], [49]
89–2579	Cheetah	Zoo, Columbus, Ohio, USA	Stomach biopsy	I	[44], [49]
90–119	Cheetah	Zoo, Columbus, Ohio, USA	Stomach biopsy	I	ATCC 51101
Tiger 1-L	Sumatran tiger	Zoo, Germany	Stomach biopsy	I	[61]
Mac	Lion	European circus	Stomach biopsy	I	[50]
Sheeba	Lion	European circus	Stomach biopsy	I	[50]
Sheena	Lion-tiger	European circus	Stomach biopsy	II	[50]
India	Sumatran tiger	European circus	Stomach biopsy	II	[51]

a all animals were kept in captivity.

### Total Protein Profiling and Expression of Homologous Pathogenicity Factors from *H. pylori*

**Figure 5:**
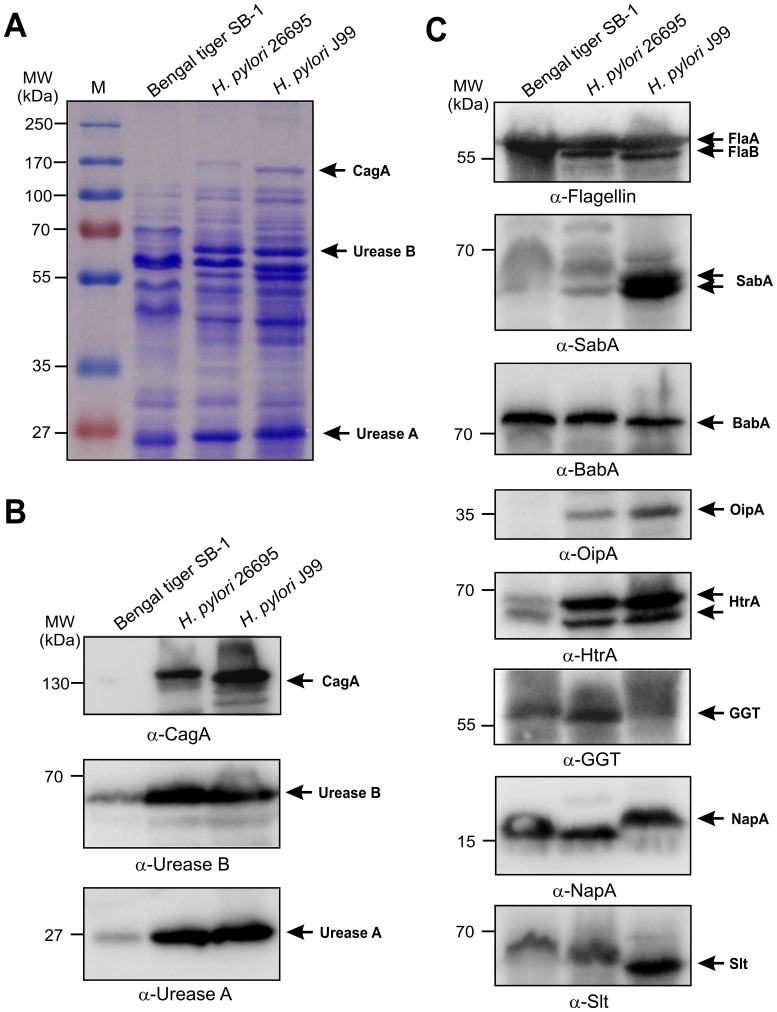
Total protein profiling and Western blotting analysis of the Bengal tiger isolate for well-known pathogenicity-associated factors reported in *H. pylori*. Panel A: Total proteins were isolated from the tiger isolate SB-1 and *H. pylori* strains 26695 and J99, separated by SDS-PAGE and stained with Coomassie Blue. The protein profiles of both 26695 and J99 strains revealed the typical *H. pylori* patterns with strong bands visible for CagA and the two major urease subunits A and B as indicated with arrows. Panel B: Western blotting analysis using *H. pylori*-specific antibodies against the effector protein CagA, and the two major urease subunits, UreA and UreB. Panel C: Detection of typical *H. pylori* flagellins (FlaA, FlaB), adhesins (BabA, SabA and OipA) and other virulence or pathogenicity-associated factors (HtrA, γ-GGT, NapA, and Slt) by Western blotting.

The close relatedness of our *H. acinonychis* isolates with *H. pylori* allowed us to screen for the presence or absence of well-known colonization and pathogenicity factors by immunoblotting. First, we compared the total protein profiles from our Bengal tiger isolate SB-1 with that of the fully-sequenced *H. pylori* strains 26695 and J99. Coomassie-blue staining of separated total proteins revealed the presence of typical bands with sizes matching those of urease subunits A and B (Fig. 5A), whereas a band in the size range of CagA (ca. 130–150 kDa) was only observed in the *H. pylori* strains, but not in SB-1 (Fig. 5A), in agreement with the PCR results described above. Second, Western blot analysis also confirmed the presence of urease A (ca. 30 kDa) and urease B (ca. 60 kDa) subunits and the absence of a CagA band in SB-1 extracts (Fig. 5B). In agreement with the finding of flagella by electron microscopy, we also found a ∼60 kDa flagellin component recognised by an *H. pylori*-specific anti-flagellin antibody in extracts of our strains (Fig. 5C, top).

The availability of antibodies against a series of other *H. pylori* proteins led us to screen more systematically for several adhesins (BabA, SabA or OipA), other virulence factors (NapA, HtrA, Slt, DupA), *cag*PAI–encoded proteins (VirB10/CagY, Cag3/Cagδ, CagM or CagN) and DNA transfer proteins (the VirD2 orthologs, Rlx1 and Rlx2) [19], [20], [62]. All antibodies were proven to specifically recognise the corresponding proteins in the *H. pylori* strains 26695 and J99, respectively (Fig. 5C and Fig. S7A–B), and our recently genome-sequenced *H. pylori* strains Shi470 and Cuz20 (accession numbers NC010698.2 and CP002076.1), which, unlike 26695 and J99, do encode full-length DupA (∼80 kDa) and Rlx (∼70 kDa) proteins (Fig. S7B). We found that while no SabA, OipA, DupA and none of the *cag*PAI proteins are expressed in *H. acinonychis*, bands corresponding in size to BabA, GGT, HtrA, NapA and Slt were produced, indicating that SB-1 contains genes for these proteins (Fig. 5C and Fig. S7A–B).

## Discussion

Numerous *Helicobacter* species have been identified in a wide range of mammals, possibly reflecting long evolutionary co-existence [1], [2], [32], [38]. The main hosts of *H. pylori* are humans and non-human primates, although this species has also been isolated from domestic cats [2], [35]–[37], and laboratory experiments have shown that it can also infect rodents (mice and gerbils), dogs, cats and pigs [1], [2]. The stomachs of mammalian carnivores (e.g. cats, dogs, lions and cheetahs) are often naturally infected by non-pylori *Helicobacter* species, including *H. felis, H. bizzozeronii* and *H. salomonis,* which are very different from *H. pylori* [1], [2], [38], and interestingly, the stomachs of large felines, can also be infected with *H. acinonychis*, which is closely related to *H. pylori* [31], [33], [44], [48]–[52], [61], [63]. However, compared to *H. pylori* we know very little about *H. acinonychis*, with most of our knowledge about its genetics deriving from the genome sequence of only one strain, Sheeba [31]. The many fragmented genes in this strain Sheeba, which are functional in *H. pylori*, led to the proposal that *H. acinonychis* was separated from *H. pylori* lineages some 200,000 years ago, perhaps after a large feline ate an *H. pylori*-infected human, thereby allowing a jump between mammalian hosts [31]. *H. acinonychis* has been isolated from captive American and European lions and cheetahs suffering from chronic gastritis and vomiting [33], [44], [63], as well as tigers and one lion-tiger hybrid [33], [61]. In the present report, we have isolated for the first time live *H. acinonychis* from diarrheic feces of a Bengal tiger (*Panthera tigris tigris*) from Thailand. Although usually *Helicobacters* have not been culturable from normal feces, our data are in accord with the success in culturing *H. pylori* from feces from humans with diarrhea [64], [65] or *H. mustelae* in feces from ferrets suffering from hypochlorhydria [66], [67].

We characterised five individual strains at the molecular level. Electron microscopy revealed a typical spiral-shaped *Helicobacter-*like organism with 1–4 monopolar sheathed or non-sheated flagella. PCR amplification, sequencing and phylogenetic analyses based on similarity values of the 16S rRNA and 23S rRNA genes identified our Bengal tiger strains as *H. acinonychis*. Prophage-specific PCR and RAPD fingerprinting confirmed that these new strains are very closely related to other *H. acinonychis* isolates. The very low diversity of *H. acinonychis* strains from different animals (tigers, cheetahs, lions and lion-tiger hybrid) and geographic origins (US, Europe and Asia), which have been placed in only two subgroups (I and II) with highly similar RAPD fingerprints, is remarkable, given that *H. pylori* isolates are extremely diverse [26], [27], [68]. The reason for this difference between the two species in strain diversity is unknown, but may reflect the relatively younger age of *H. acinonychis* as a species, or evolutionary constraints imposed in its special big cat hosts.

The availability of *H. pylori* specific antibodies enabled us for the first time to screen *H. acinonychis* for the expression of certain pathogenicity associated proteins by Western blotting. Our data indicate that Bengal tiger isolates express a flagellin (∼60 kDa), but the flagella structures seen by electron microscopy were morphologically distinct from that of *H. pylori*. Another factor important for colonisation in the stomach is the urease complex, which consists of two major subunits (UreA and UreB) and some accessory proteins. Urease activity neutralising gastric pH is required to survive in an acid milieu, and also may play a role in *H. pylori* metabolism [5], disruption of transepithelial resistance [69] and pro-inflammatory responses [70]. Both proteins are highly conserved, bands corresponding to both UreA and UreB proteins were expressed, and functional assays demonstrated a highly active urease complex in our *H. acinonychis* isolates.

Adherence of *H. pylori* to specific glycan receptors in the human gastric mucosa by outer membrane protein family members BabA, SabA and OipA adhesins is widely assumed to be adaptive, to contribute importantly to initial colonization and long-term persistence in human hosts [6]–[8], [71], [72]. In contrast, the potential adhesins of *H. acinonychis* are completely unknown. In agreement with the host jump theory, the frequency of fragmented genes is particularly high in the sequenced *H. acinonychis* Sheeba strain as compared to *H. pylori* genomes, and this includes 12 OMPs, VacA and others [31]. Thus, it is probably not surprising that we could not detect a Western blot band specific for SabA in *H. acinonychis*. Interestingly, we observed a ∼72 kDa band reacting with BabA antiserum in *H. acinonychis*. A full-length ortholog of BabA has not been noted in the Sheeba genome [31], but some of the OMPs in the Sheeba strain exhibit homologous stretches to BabA, which may explain our Western blot results. Furthermore, no protein band corresponding to OipA was found in *H. acinonychis*, which is in line with the observation that one gene in the Sheeba genome (OMP-7, fragment 2), showing extensive homology to OipA, is fragmented and therefore unlikely to be expressed in *H. acinonychis*.

In agreement with the absence of *cag*PAI and *cagA* genes in previously analysed *H. acinonychis* isolates [31], [33], we were also unable to detect any protein expression for CagA and well-known other *cag*PAI components such as Cagδ, CagM, CagN or VirB10. Furthermore, we also failed to PCR amplify conserved fragments of *vacA, cagA* and other *cag*PAI genes. However, we were able to detect full-length proteins in *H. acinonychis* for a series of other well-known *H. pylori* pathogenicity factors including NapA, GGT, HtrA and Slt. The detection of GGT, NapA and Slt may explain the chronic gastritis as characterized by the occurrence of inflammatory cells in the gastric mucosa observed of some infected felines [44], [49], [61]. The finding of an HtrA ortholog in *H. acinonychis* also raises the possibility that this protein may disturb epithelial barrier functions by cleaving E-cadherin [17], [18], [73], which also could be involved in the gastric pathology observed in big cats.

In *H. pylori* there recently has been considerable interest in strain-specific genes in the so-called plasticity regions, which are large, possibly conjugative, transposons or transposon remnants [74]. Recent work has shown that they encode putative pathogenicity factors, such as the duodenal ulcer-promoting gene A (*dupA*), which has been associated with duodenal ulceration [13]. It has been noted that extensive size variation exists in the *dupA* genes among clinical *H. pylori* isolates, which may interfere with their putative activity [14], . Other genes include those encoding putative DNA transfer enzymes, such as the relaxases Rlx1 and Rlx2 [19], [20], [62]. Interestingly, in *H. acinonychis* we found proteins of similar size to those of *H. pylori* that cross-reacted with antisera to both Rlx1 and Rlx2, but no cross-reactivity with an anti-DupA antibody. The role of Rlx1 and Rlx2 is not fully clear, but they may be involved in the exchange of genetic material between bacteria, which warrant further investigations [19], [20], [62], [74].

Taken together, we have morphologically and genetically characterised new *Helicobacter* spp. strains isolated from a Bengal tiger in Thailand, which were classified as *H. acinonychis* and which show similar genetic background to those of previously isolated *H. acinonychis* strains from captive tigers, lions and cheetahs located in Europe or the US. Currently there is only one *H. acinonychis* genome sequence available, strain Sheeba, isolated from a lion housed in a Russian circus [31], and there is a lack of other genetic information in databases referring to different *H. acinonychis* strains isolated from big cats located in various geographic locations. Thus, this is the first report of an *H. acinonychis* isolate from an Asian tiger. In addition, we have shown remarkably similar RAPD fingerprinting patterns between worldwide *H. acinonychis* isolates and screened for known pathogenicity factors from *H. pylori* such as flagellin, ureaseA/B, NapA, HtrA, GGT, Slt, two relaxases and probably a BabA-like protein. Most of these genes are expressed in *H. acinonychis* strains isolated from the Bengal tiger. However, CagA and *cag*PAI factors, as well as VacA, OipA, SabA and DupA, were not detected. An important challenge for the future will be to identify the function of proteins involved in colonization and disease development. The use of mouse-adapted *H. acinonychis* strains [33] should be a valuable approach for analyzing the interplay between this human-derived animal pathogen and its host. These studies could reveal the specificity of infections and help us understand the evolutionary routes used by these gastric pathogens.

## Materials and Methods

### Bacterial Isolation

Colonies of *Helicobacter* strains were isolated from diarrheic feces of a captive Bengal tiger (*Panthera tigris tigris*) suffering from gastritis in a zoo in Bangkok/Thailand. The samples were collected in sterile tubes, incubated with brain heart infusion (BHI) medium (5 mL per gram material), shaken for 20 min at 37°C in 50 mL Falcon tubes at 1,000×*g*. The mixture was then centrifuged for 10 min at 2,000×*g* to remove larger particles and non-digested material. The supernatant was removed and passed through sterile filter paper (Whatman, GE Healthcare, UK limited Amersham Place, UK) to further remove debris. Bacteria were then cultured in different amounts (100, 50, 25 or 5 µL) on different agar plates (*H. pylori* selective agar plates, GC agar plates with 10% horse serum, *Campylobacter* selective plates, Müller-Hinton agar plates, and Columbia agar plates containing 5% sheep blood). These plates were incubated for 2, 3, 4, and 7 days, respectively. The gas generating systems Campygen, Anaerogen (both from Oxoid/Fisher Scientific, Germany), Anaerocult (Merck, Darmstadt, Germany), and an anaerobic chamber with a mix of nitrogen, carbon dioxide and hydrogen (90%, 5% and 5%, respectively) were used for incubation at 37°C. Single bacterial colonies (called SB-1, SB-2, SB-3, SB-4 and SB-5) were isolated and grown on Columbia agar plates containing 5% sheep blood and Campygen for further analyses.

### Gram-staining

Grown bacterial colonies were screened by standard Gram-staining (Crystal violet, Gram’s iodine solution, acetone/ethanol (50∶50 vol/vol), 0.1% basic fuchsin solution) [47]. This method was applied as an initial step to investigate the morphology, homogeneity and culture purity of the isolated bacterial microorganisms.

### Bacterial Strains and Culture Conditions

We included in our studies some other described *Helicobacter* species as controls: *H. acinonychis* (ATCC51101), *H. felis* (ATCC49179), *H. fennelliae* (ATCC35684), *H. hepaticus* strain 1549/00 [47], *H. mustelae* (strain NL03-2004, unpublished), *H. salomonis* (strain NL07-2005, unpublished), *H. bilis* (ATCC43879), *H. cinaedi* (DSM5359, DSMZ Braunschweig, Germany), *H. typhlonius* (MIT 97-6810), *H. magdeburgensis* (strain HM-007) [47], *H. bizzozeronii* (strain NL07-2005, unpublished), *H. canis* (ATCC51401) and *H. aurati* (MIT 97-5075). In addition, we included the four fully sequenced *H. pylori* strains 26695, J99 [28], Cuz20 and Shi470 [75], and a series of *H. acinonychis* strains (Table 1) including two other widely uncharacterised isolates from a Sumatran tiger, *Panthera tigris sumatrae* [61]. All strains were grown under standard conditions as described. To test for functional urease activity, bacteria were grown on selective acidified agar plates supplemented with urea, the substrate of *H. pylori* urease, and phenol red as indicator [55].

### Ethics Statement

The fecal samples were collected with permission and help by a zookeeper. An Ethics statement was not necessary as the sample was not collected by an invasive method disturbing the tiger in any aspect. The tiger was actually not affected in any way nor harmed.

### DNA Isolation, PCR Analyses and Sequencing

Plate-grown *Helicobacter* sp. were harvested with a sterile cotton swab and suspended in 200 µL of lysis buffer (50 mM Tris-HCl (pH 7.6), 100 mM EDTA, 0.5% Tween-20, 20 mg of proteinase K per mL) and incubated at 58°C for 2 hours [47]. The proteinase K was inactivated by conventional phenol/chloroform extraction method. Purified DNA was then precipitated with 2.5 volume of 96% ethanol and washed with 70% ethanol. To investigate the presence of certain genes, we performed PCR assays using the primers summarised in Table S1. DNA sequences were determined by standard sequencing procedures [47], [76]. The following gene sequences from strain SB-1 were deposited in GenBank databases: 16S rRNA (accession number JN251811.1), 23S rRNA (KC470072.1 and KC470071.1), flagellin (KC470069.1), urease (KC470068.1) and helicase (KC470070.1). Sequence comparison was performed using NCBI database tools (http://blast.ncbi.nlm.nih.gov/).

### 16S and 23S rRNA Sequence Analyses

16S and 23S rRNA sequence data from the tiger strain and from closely related species, collected from GenBank, were aligned using BioEdit [77]. Aligned sequences were then imported as FASTA to MEGA5 [78] to determine DNA relatedness using the Neighbor-Joining method [79] and to construct the optimal tree using the Maximum Composite Likelihood method [80].

### Restriction Fragment Length Polymorphism (RFLP) of the 16S rRNA Gene

For restriction fragment analysis of the 16S rRNA gene, we amplified by PCR a specific and conserved 1.2 kb subfragment as described [53] (Table S1). RFLP patterns of amplified PCR products were obtained with each of the following restriction enzymes, *Alu*I and *Hha*I [54], [81]. Digests were performed in the appropriate 1×buffers as recommended by the manufacturer (New England Biolabs, Acton, MA, USA).

### Randomly Amplified Polymorphic DNA (RAPD) Fingerprinting PCR

The RAPD fingerprinting method established to study *H. pylori* strains [27], was used to compare the diversity of the DNA sequences among the *Helicobacter* strains tested. This method uses arbitrary oligonucleotide sequences to prime DNA fragments from the whole genome. We used 20 ng genomic DNA from each strain as template, 20 pmol of each primer (Table S1), 1U Taq DNA-polymerase (Qiagen, Hilden, Germany) and 250 µM from each dNTP, 1×buffer, and sterilized double distilled water for a total volume of 50 µL. A Perkin-Elmer thermal cycler model 9700 was used for amplification reactions. The cycling program was four cycles of 94°C, 5 min; 40°C, 5 min; 72°C, 5 min; low stringency amplification, and a final incubation at 72°C for 10 min.

### Field Emission Scanning Electron Microscopy (FESEM)

Bacterial cells were harvested and fixed in a sterile solution containing 5% formaldehyde, 2% glutaraldehyde in cacodylate buffer (0.1 mM cacodylate, 0.01 mM CaCl_2_, 0.01 mM MgCl_2_, 0.09 mM sucrose, pH 6.9) for 1 hour on ice [82], [83]. The solution was centrifuged and passed through a sterile filter. After several washes with cacodylate buffer and TE buffer (20 mM Tris, 1 mM EDTA, pH 6.9), samples were dehydrated in serial dilutions of acetone (10%, 30%, 50%, 70%, 90%, and 100%) on ice for 15 min each step. Samples were then allowed to reach room temperature before another change of 100% acetone, after which they were subjected to critical-point drying with liquid CO_2_ (CPD030; Bal-Tec, now Leica, Wetzlar, Germany). Samples were finally covered with a ca. 10.0 nm thick gold film by sputter coating (SCD500; Bal-Tec) and examined in a field emission scanning electron microscope (Zeiss DSM 982 Gemini) using an Everhart Thornley SE detector and in-lens detector in a 50∶50 ratio at an acceleration voltage of 5.0 kV.

### Electron Microscopic Analysis by Negative Staining

For negative staining, thin carbon support films were prepared by indirect sublimation of carbon on freshly cleaved mica. Samples were then absorbed to the carbon film and negatively stained with 1% (wt/vol) aqueous uranyl acetate (pH 4.5). After air drying, samples were examined by transmission electron microscopy (TEM) in a Zeiss TEM 910 at an acceleration voltage of 80 kV and at calibrated magnifications using a line grid replica. Images were recorded digitally with a Slow-Scan CCD-Camera (ProScan, 1024×1024, Scheuring, Germany) with ITEM-Software (Olympus Soft Imaging Solutions, Münster, Germany).

### Protein Profiling

A previously published method was adapted [84]. Briefly, a washed pellet of the strains *Hp* 26695, J99, Shi470 or Cuz20 and Bengal tiger isolate was suspended in 0.5 mL of sodium dodecyl sulfate (SDS) buffer (50 mM Tris hydrochloride (pH 6.8), 5% β-mercaptoethanol (vol/vol), 1% sodium dodecyl sulphate (wt/vol), 15% glycerol (vol/vol), and 0.01% bromophenol blue). The homogenate was heated for 5 min at 95°C. Insoluble debris was removed by centrifugation at 10,000×g for 5 min. Supernatants were subjected to 6% and 10% SDS polyacrylamide gel electrophoresis (SDS-PAGE) gels and blotted by Semi dry blotting.

### Antibodies and Immunoblotting Analyses

The following primary antibodies were used: Rabbit polyclonal anti-CagA antibody was purchased from Austral Biological (San Ramon, CA, USA). The mouse polyclonal anti-urease antibodies and anti-CagN antibodies were described elsewhere [85], [86]. Polyclonal rabbit antibodies recognizing a series of other *H. pylori* proteins, were raised against peptides corresponding to the following conserved amino acid (aa) residues derived from strain 26695: BabA (aa 126–140: CGGNANGQESTSSTT), SabA (aa 172–186: CAMDQTTYDKMKKLA), OipA (aa 275–282: NYYSDDYGDKLDYK), NapA (aa 105–118: EFKELSNTAEKEGD), Slt (aa 492–505: LRRWLESSKRFKEK), HtrA (aa 90–103:DKIKVTIPGSNKEY), FlaA (aa 93–106: KVKATQAAQDGQTT), GGT (aa 175–188: RQAETLKEARERFL), DupA (aa 551–564: MLNIDSDNQQDNKA), VirB10/CagY (repeat region: VSRARNEKEKKE), Cagδ (aa 32–45: IKATKETKETKKEA), and Rlx2 (aa 131–144: HLVFSIDENSNEKN). Rabbit anti-Rlx1 and anti-CagM antibodies were raised against the entire recombinant Rlx1 or CagM proteins, respectively. All antibodies were affinity-purified and prepared according to standard protocols by Biogenes GmbH (Berlin, Germany). Horseradish peroxidase-conjugated anti-mouse or anti-rabbit polyvalent sheep immunglobulin was used as secondary antibody (DAKO Denmark A/S, DK-2600 Glostrup, Denmark) and blots were developed with ECL Plus Western blot reagents (GE Healthcare, UK limited Amersham Place, UK) [87]–[89].

## Supporting Information

Figure S1 **Morphological analyses of novel** ***Helicobacters*** **from a Bengal tiger by scanning electron microscopy.** The majority of bacteria contained either no or 1–4 monopolar sheated flagella as shown. Representative pictures are shown from two preparations. Each bar corresponds to 1 µm.
(PDF)

Figure S2 **Analysis of 16SrRNA by RFLP and RAPD fingerprinting of different** ***Helicobacter*** **species.** Panel A: Schematic representation of the 1.2 kb 16S rRNA gene PCR product with indicated restriction sites for endonuclease *Hha*I. Panel B: DNA isolated from various *Helicobacter* species, including the Bengal tiger isolate SB-1, was amplified followed by RFLP using *Hha*I. Similar bands were obtained in the RFLP pattern of *H. pylori*, *H. acinonychis*, *H. mustelae*, *H. bilis*, *H. magdeburgensis* and *H. canis* (lanes marked with asterisks), indicating their close genetic relatedness. Panels C/D: RAPD fingerprinting of the *Helicobacter* isolates using primer D-9355 (panel C) and D-8635 (panel D) was performed as described [27]. Typical RAPD fingerprinting profiles are shown and revealed the relatedness between *H. pylori*, *H. acinonychis* and SB-1. M, DNA size marker.
(PDF)

Figure S3 **Phylogenetic tree of the 23S ribosomal RNA gene from the tiger strain SB-1 and the most closely related sequences from different** ***Helicobacter*** **species.** The alignment was performed with BioEdit using gap penalties of 10 for gap opening, 5 for gap extension and a bootstrap value of 1,000. MEGA5 was used to infer DNA relatedness using the Neighbor-Joining method. The evolutionary distances were computed using the Maximum Composite Likelihood method and are in the units of the number of base substitutions per site. The optimal tree with the sum of branch length was equal to 0.3061 for 23S rRNA. *Helicobacter* sp. and *Wolinella succinogenes* were used as outgroups. The phylogenetic tree shows that the 23SrRNA gene of our Bengal tiger strain (accession number KC470072.1) branched together with *Helicobacter acinonychis* from a Sumatran tiger, thus demonstrating close relatedness among them.
(PDF)

Figure S4 **Analysis of individual** ***Helicobacter*** **colonies from Sumatran and a Bengal tiger by 16SrRNA PCR and RFLP.** Panel A: Schematic representation of the 1.2 kb 16S rRNA gene PCR product with indicated restriction sites for *Alu*I and *Hha*I, respectively. Panel B: A conserved 1.2 kb DNA fragment of the 16S rRNA gene in the genus *Helicobacter* [53] was amplified from two *H. acinonychis* colonies from a Sumatran tiger [59] and five colonies from a Bengal tiger investigated in this study. Panels C/D: To confirm the specificity of these fragments, all PCR products were then digested with the restriction endonucleases *Alu*I (panel B) or *Hha*I (panel C) giving rise to a specific banding pattern as described [53], and which was identical among all investigated clones indicating their close genetic relatedness.
(PDF)

Figure S5 **Analysis of individual** ***Helicobacter*** **colonies from Sumatran and a Bengal tiger by RAPD fingerprinting.** Panel A–C: To investigate the genetic relatedness among individual colonies isolated from tigers, total DNA isolated from two *H. acinonychis* colonies from a Sumatran tiger [61] and five colonies from our Bengal tiger was subjected to RAPD fingerprinting analysis as described elsewhere [27]. This method uses a set of single primers (D-14307, D-9355 or D-8635 as shown in panels A–C) which arbitrarily anneal and amplify genomic DNA resulting in strain-specific fingerprinting patterns [27]. The RAPD patterns were highly similar but not fully identical among all investigated clones indicating their close genetic relatedness.
(PDF)

Figure S6 **Genetic relatedness of various** ***Helicobacter acinonychis*** **isolates from different big cats from the US, Europe and Asia as analysed by RAPD fingerprinting.** Panel A–C: RAPD fingerprinting PCR of the indicated *H. acinonychis* isolates from tigers, cheetahs, lions and lion-tiger (compare Fig. 4C and Table 1) reveals the close relatedness between strains in two specific groups, called I and II, as indicated. The RAPD primers D-1281, D-1283 and D-1290 [27] have been used in this experiment and are shown in panels A, B and C, respectively. M, DNA size marker.
(PDF)

Figure S7 **Western blotting analysis of the Bengal tiger isolate SB-1 for well-known pathogenicity-associated factors reported in** ***H. pylori*** **.** Panel A: Total proteins were isolate from SB-1 and *H. pylori* strains 26695 and J99, separated by SDS-PAGE and stained with the indicated antibodies against typical *H. pylori* proteins of the *cag* type IV secretion system, showing their presence in both *H. pylori* strains but absence in SB-1. Panel B: Total proteins were isolated from SB-1 and *H. pylori* strains Cuz20 and Shi470, separated by SDS-PAGE and stained with the indicated antibodies against two potential DNA transfer proteins (relaxases), Rlx1 and Rlx2 [19], [20], and the duodenal ulcer promoting gene A (DupA). Both *H. pylori* strains express all three proteins, while SB-1 only exhibits a band for Rlx1 and Rlx2, but not DupA.
(PDF)

Table S1 (DOC)
