# Supplementary figures and images for: Electron Microscopic, Genetic and Protein Expression Analyses of Helicobacter acinonychis Strains from a Bengal Tiger
Source: PLoS One. 2013 Aug 5;8(8):e71220. doi: 10.1371/journal.pone.0071220 (PMC3733902; doi:10.1371/journal.pone.0071220)

**Figure S1**

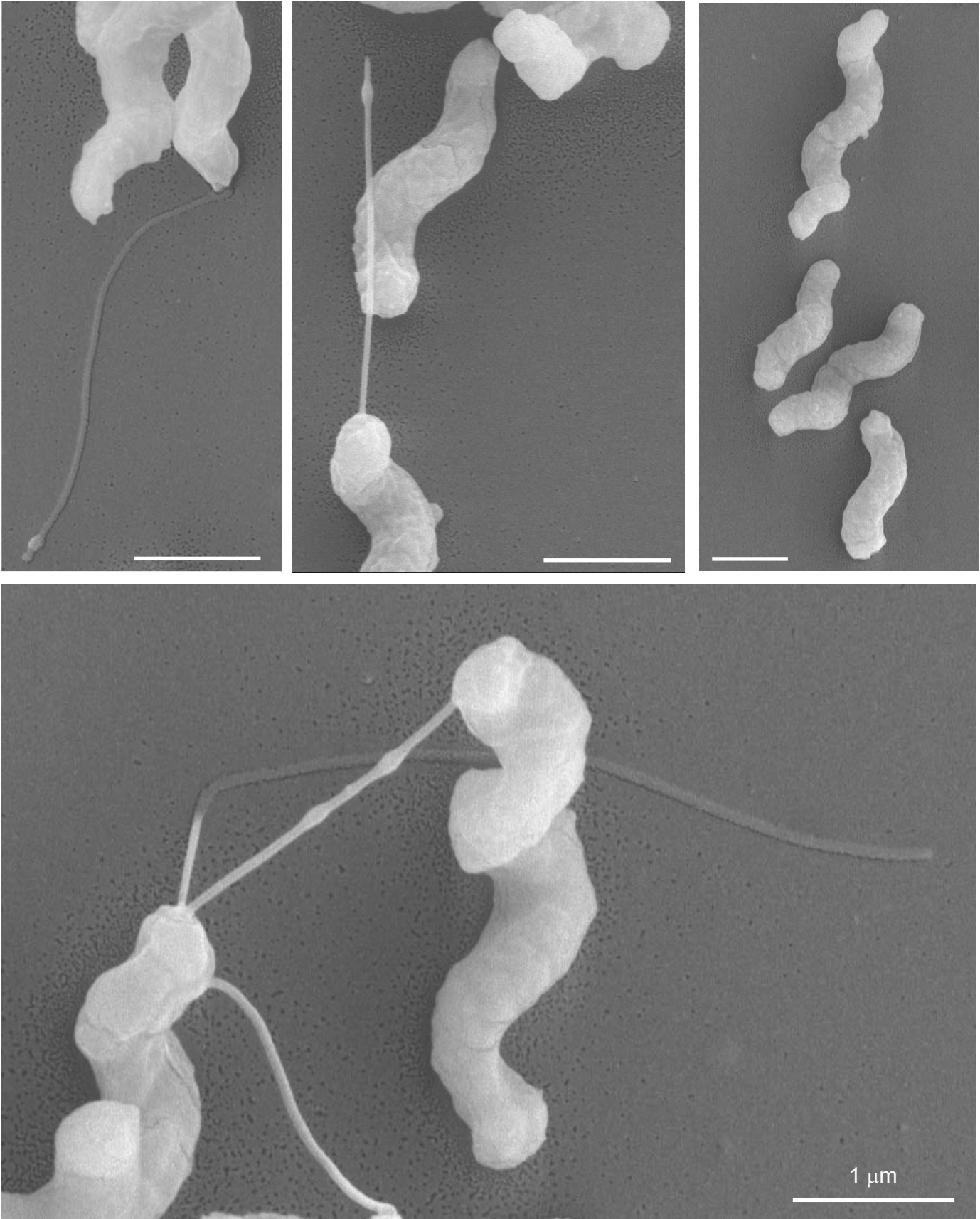

Supplement: Figure S1 — Morphological analyses of novel Helicobacters from a Bengal tiger by scanning electron microscopy. The majority of bacteria contained either no or 1–4 monopolar sheated flagella as shown. Representative pictures are shown from two preparations. Each bar corresponds to 1 µm. (PDF) [file pone.0071220.s001.pdf]

**Figure S2**

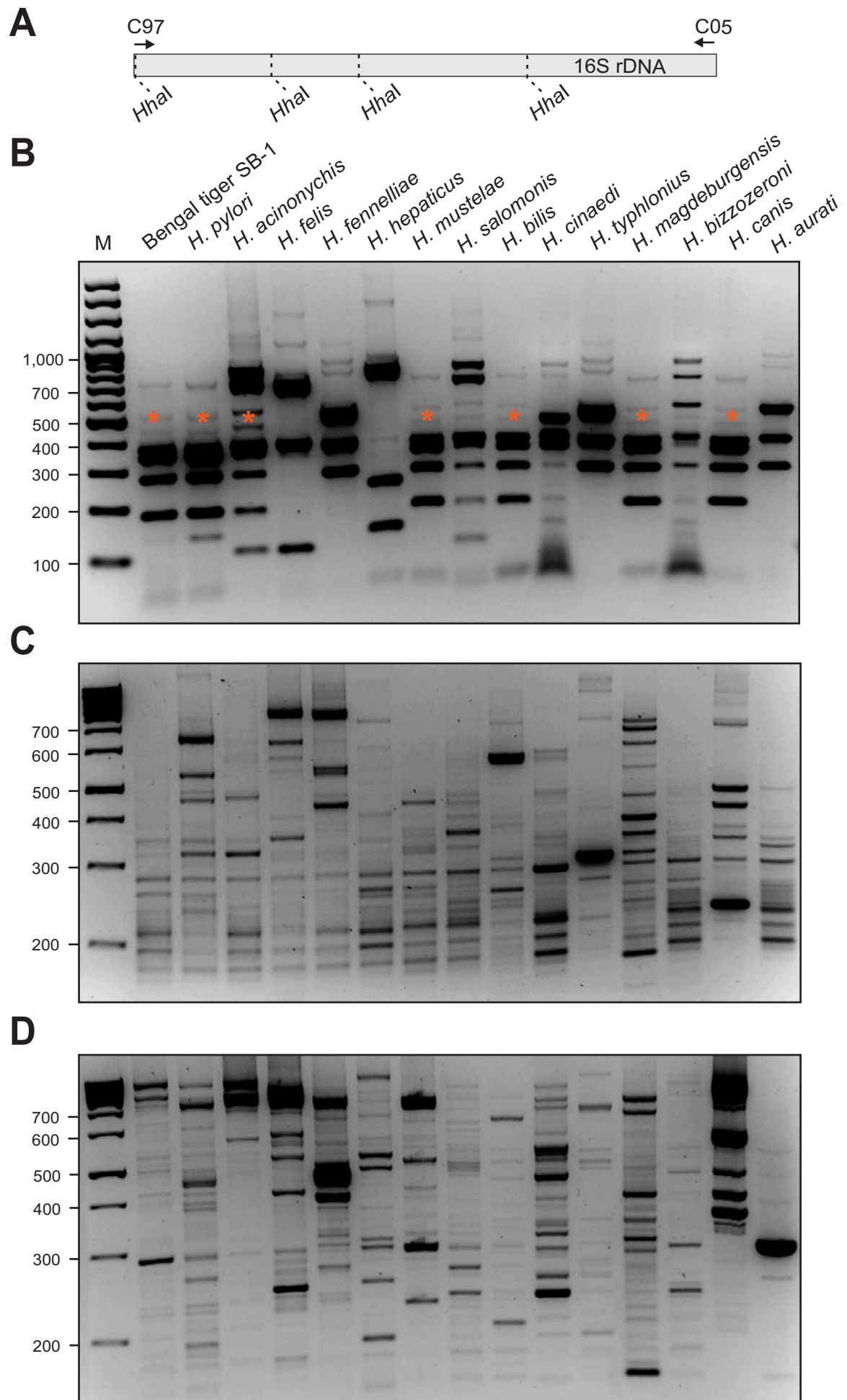

Supplement: Figure S2 — Analysis of 16SrRNA by RFLP and RAPD fingerprinting of different Helicobacter species. Panel A: Schematic representation of the 1.2 kb 16S rRNA gene PCR product with indicated restriction sites for endonuclease HhaI. Panel B: DNA isolated from various Helicobacter species, including the Bengal tiger isolate SB-1, was amplified followed by RFLP using HhaI. Similar bands were obtained in the RFLP pattern of H. pylori, H. acinonychis, H. mustelae, H. bilis, H. magdeburgensis and H. canis (lanes marked with asterisks), indicating their close genetic relatedness. Panels C/D: RAPD fingerprinting of the Helicobacter isolates using primer D-9355 (panel C) and D-8635 (panel D) was performed as described [27]. Typical RAPD fingerprinting profiles are shown and revealed the relatedness between H. pylori, H. acinonychis and SB-1. M, DNA size marker. (PDF) [file pone.0071220.s002.pdf]

Figure S3

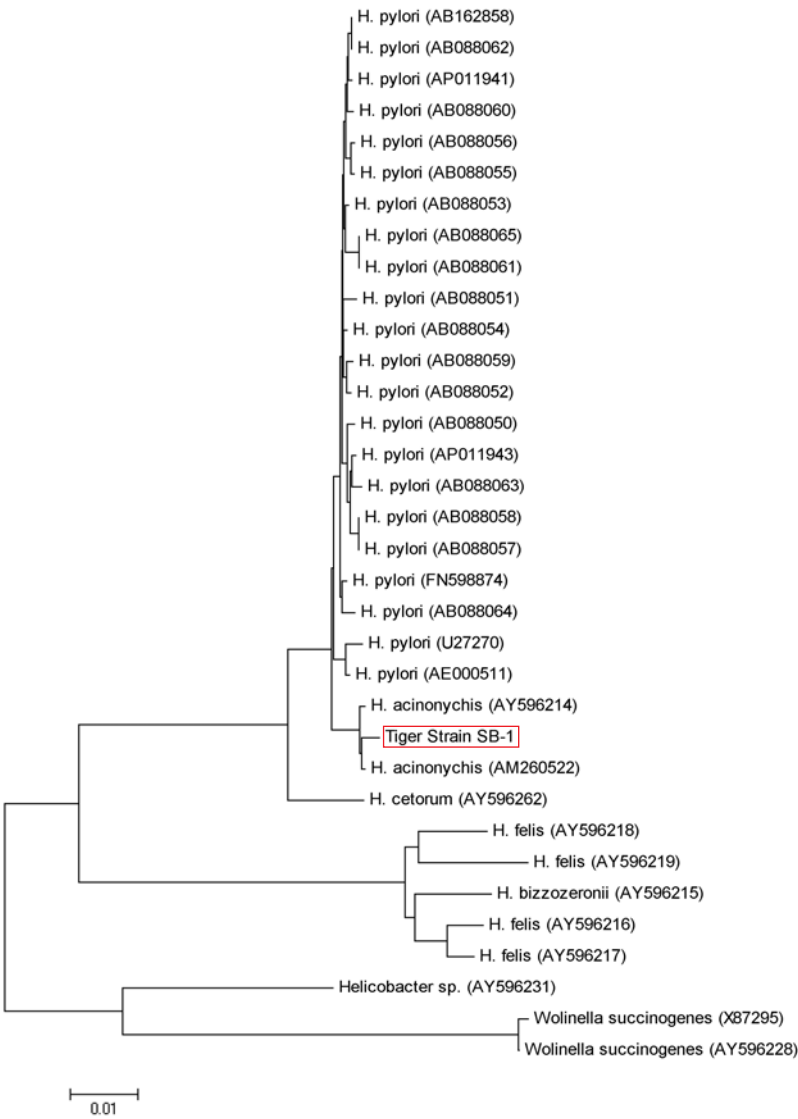

Supplement: Figure S3 — Phylogenetic tree of the 23S ribosomal RNA gene from the tiger strain SB-1 and the most closely related sequences from different Helicobacter species. The alignment was performed with BioEdit using gap penalties of 10 for gap opening, 5 for gap extension and a bootstrap value of 1,000. MEGA5 was used to infer DNA relatedness using the Neighbor-Joining method. The evolutionary distances were computed using the Maximum Composite Likelihood method and are in the units of the number of base substitutions per site. The optimal tree with the sum of branch length was equal to 0.3061 for 23S rRNA. Helicobacter sp. and Wolinella succinogenes were used as outgroups. The phylogenetic tree shows that the 23SrRNA gene of our Bengal tiger strain (accession number KC470072.1) branched together with Helicobacter acinonychis from a Sumatran tiger, thus demonstrating close relatedness among them. (PDF) [file pone.0071220.s003.pdf]

Figure S4

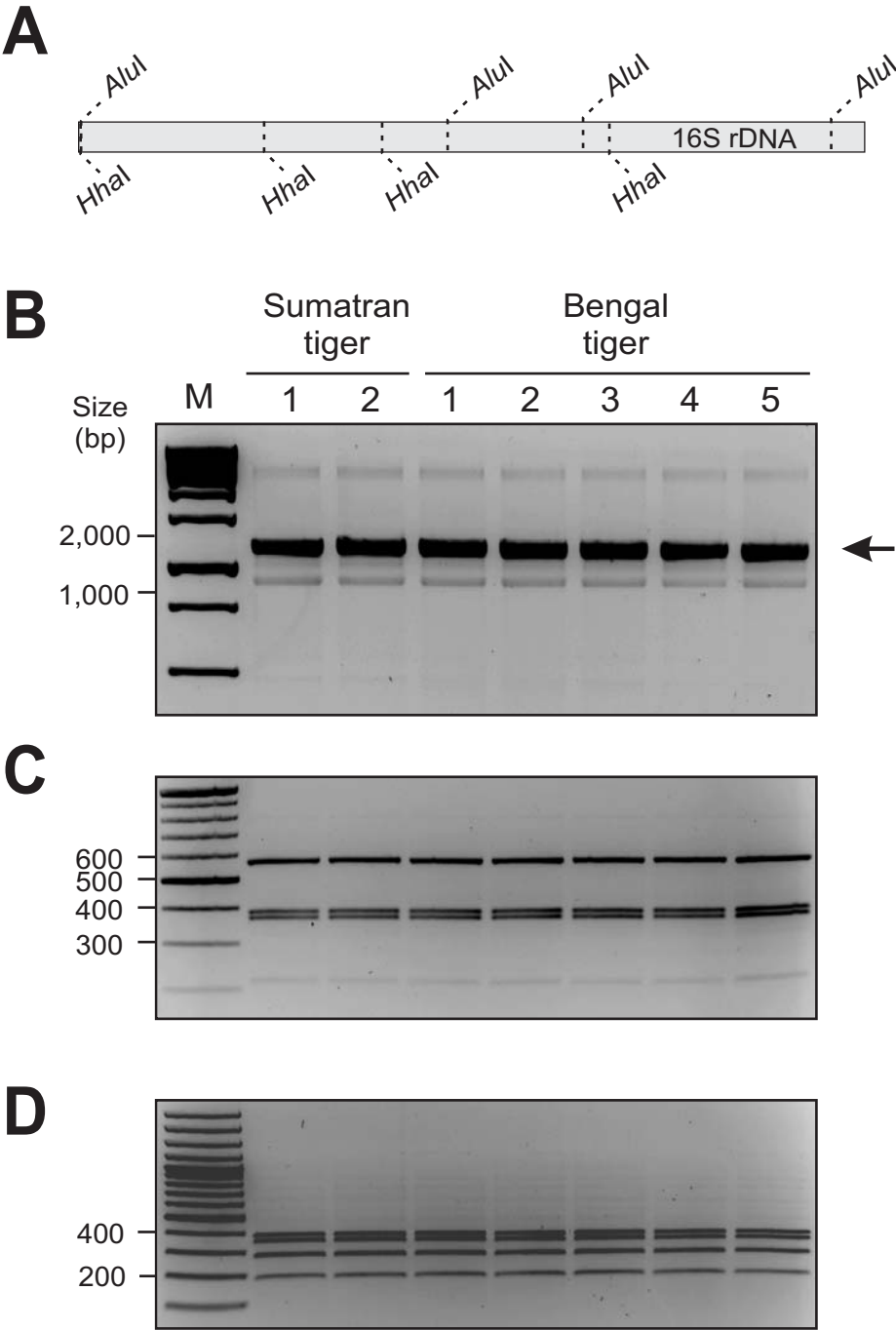

Supplement: Figure S4 — Analysis of individual Helicobacter colonies from Sumatran and a Bengal tiger by 16SrRNA PCR and RFLP. Panel A: Schematic representation of the 1.2 kb 16S rRNA gene PCR product with indicated restriction sites for AluI and HhaI, respectively. Panel B: A conserved 1.2 kb DNA fragment of the 16S rRNA gene in the genus Helicobacter [53] was amplified from two H. acinonychis colonies from a Sumatran tiger [59] and five colonies from a Bengal tiger investigated in this study. Panels C/D: To confirm the specificity of these fragments, all PCR products were then digested with the restriction endonucleases AluI (panel B) or HhaI (panel C) giving rise to a specific banding pattern as described [53], and which was identical among all investigated clones indicating their close genetic relatedness. (PDF) [file pone.0071220.s004.pdf]

Figure S5

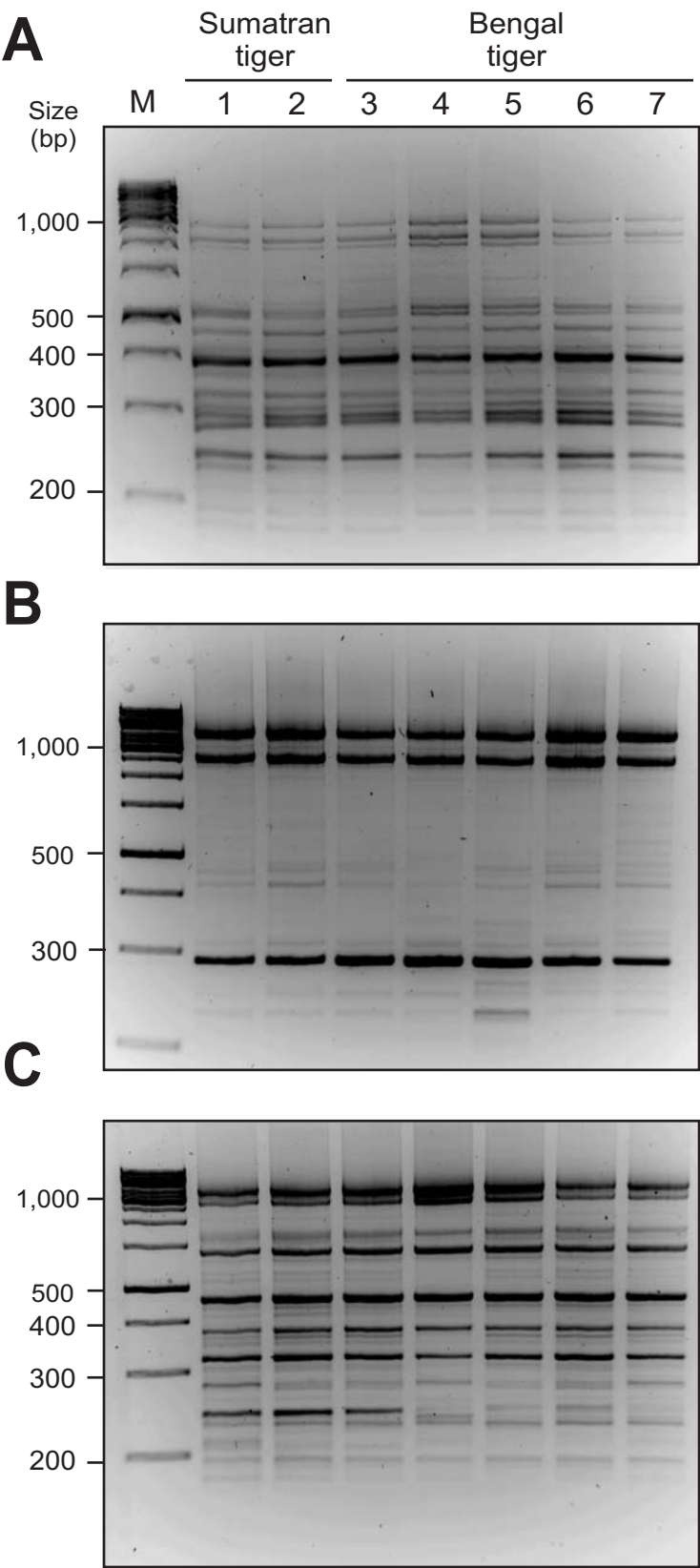

Supplement: Figure S5 — Analysis of individual Helicobacter colonies from Sumatran and a Bengal tiger by RAPD fingerprinting. Panel A–C: To investigate the genetic relatedness among individual colonies isolated from tigers, total DNA isolated from two H. acinonychis colonies from a Sumatran tiger [61] and five colonies from our Bengal tiger was subjected to RAPD fingerprinting analysis as described elsewhere [27]. This method uses a set of single primers (D-14307, D-9355 or D-8635 as shown in panels A–C) which arbitrarily anneal and amplify genomic DNA resulting in strain-specific fingerprinting patterns [27]. The RAPD patterns were highly similar but not fully identical among all investigated clones indicating their close genetic relatedness. (PDF) [file pone.0071220.s005.pdf]

Figure S6

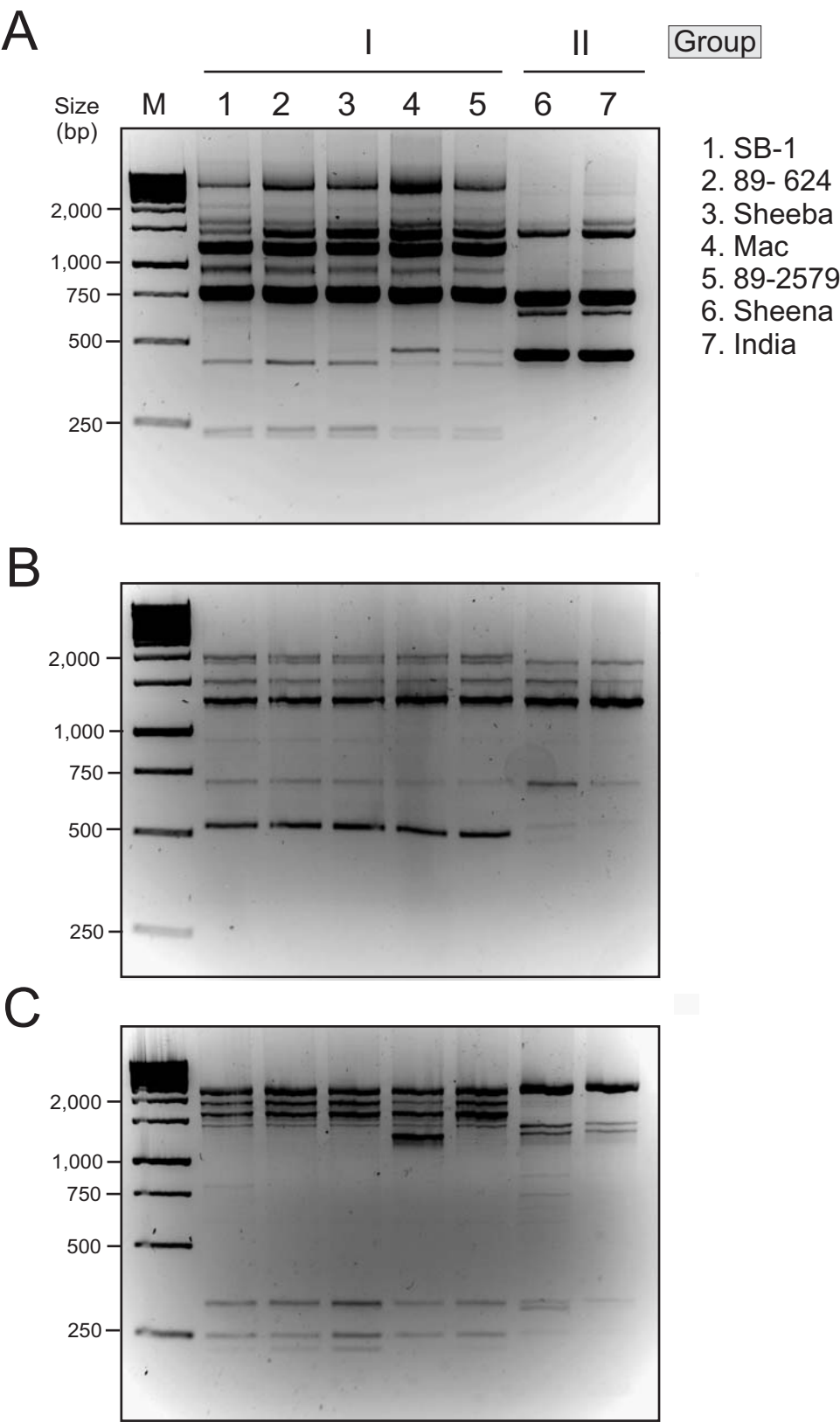

Supplement: Figure S6 — Genetic relatedness of various Helicobacter acinonychis isolates from different big cats from the US, Europe and Asia as analysed by RAPD fingerprinting. Panel A–C: RAPD fingerprinting PCR of the indicated H. acinonychis isolates from tigers, cheetahs, lions and lion-tiger (compare Fig. 4C and Table 1) reveals the close relatedness between strains in two specific groups, called I and II, as indicated. The RAPD primers D-1281, D-1283 and D-1290 [27] have been used in this experiment and are shown in panels A, B and C, respectively. M, DNA size marker. (PDF) [file pone.0071220.s006.pdf]

**Figure S7**

**A**

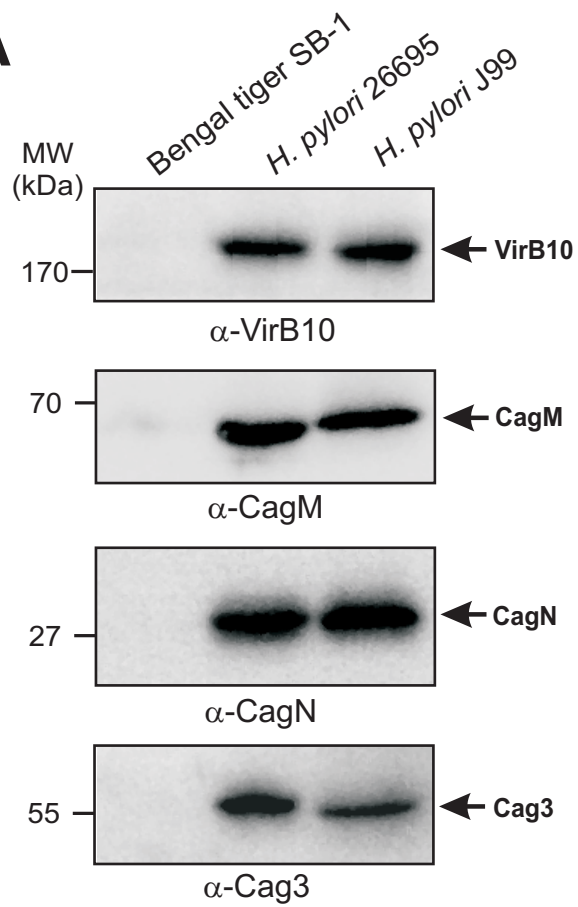

**B**

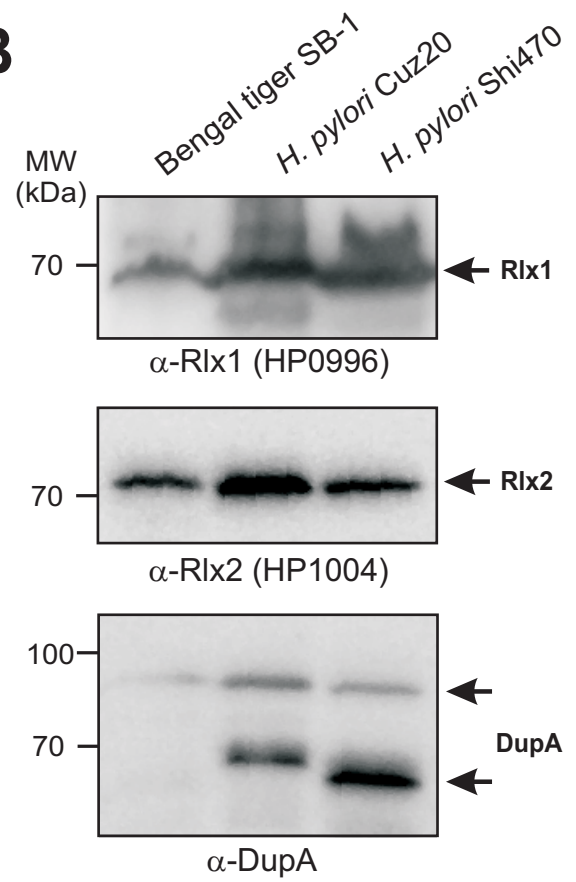

Supplement: Figure S7 — Western blotting analysis of the Bengal tiger isolate SB-1 for well-known pathogenicity-associated factors reported in H. pylori . Panel A: Total proteins were isolate from SB-1 and H. pylori strains 26695 and J99, separated by SDS-PAGE and stained with the indicated antibodies against typical H. pylori proteins of the cag type IV secretion system, showing their presence in both H. pylori strains but absence in SB-1. Panel B: Total proteins were isolated from SB-1 and H. pylori strains Cuz20 and Shi470, separated by SDS-PAGE and stained with the indicated antibodies against two potential DNA transfer proteins (relaxases), Rlx1 and Rlx2 [19], [20], and the duodenal ulcer promoting gene A (DupA). Both H. pylori strains express all three proteins, while SB-1 only exhibits a band for Rlx1 and Rlx2, but not DupA. (PDF) [file pone.0071220.s007.pdf]
