# Supplementary material for: Electron Microscopic, Genetic and Protein Expression Analyses of Helicobacter acinonychis Strains from a Bengal Tiger
Source: PLoS One. 2013 Aug 5;8(8):e71220. doi: 10.1371/journal.pone.0071220 (PMC3733902; doi:10.1371/journal.pone.0071220)
Supplement: Table S1 — (DOC) [file pone.0071220.s008.doc]

**Suppl. Table1:** Primers used for PCR and sequencing of *H. acinonychis* gene fragments.

| **Gene** | **Primer name** | **Primer sequence^a^** | **PCR fragment length** | **Reference** |
| --- | --- | --- | --- | --- |
| 16S rRNA^b^ | C97 | 5'-CGTATGACGGGTATCC | ~1,200 bp | [53] |
|  | CO5 | 5'-ACTTCACCCCAGTCGCGT |  |  |
| 23S rRNA^b^ | O68 | 5´-AGGCGATGAAGGACGTA | 1,580 bp | [90] ^e^ |
|  | M86 | 5´-CYACCTGTGWCRGTTT |  |  |
| 23S rRNA^b^ | M93 | 5´-WGCGTAAYAGCTCAC | 1,579 bp | [90] ^e^ |
|  | P46 | 5´-CGGTCCTCTCGTACTAG |  |  |
| *ureA* ^c^ | SB1F-*ureA* | 5´-CACCCCAAAAGAGTTAGACAA | 507 bp | This study |
|  | SB1R-*ureA* | 5´-GTCCCACTCGCAATATCTAAT |  |  |
| *ureB* ^c^ | SB1F-*ureB* | 5´-CGCAAAAGGTTTGTGGCGTTC | 506 bp | This study |
|  | SB1R-*ureB* | 5´-GGCGTGGTAAAAAAACGGTGG |  |  |
| *flaA* ^c^ | SB1F-*flaA* | 5´-CTTCAGCAAAATCCACATCCC | 767 bp | This study |
|  | SB1R-*flaA* | 5´-CAACAAAAACTCTAACCGAACC |  |  |
| *flaB* ^c^ | SB1F-*flaB* | 5´-CAAAAATGCCCCTAACCGCC | 1,152 bp | This study |
|  | SB1R-*flaB* | 5´-CCAATATCGCCGCTTTAACTTC |  |  |
| *helicase* ^c^ | SB1F-proΦ | 5´-GCCAGACTGAATAAGCCGAAC | 1,360 bp | This study |
|  | SB1R- proΦ | 5´-CAAAGAACAACTAAGCGAAGACAC |  |  |
| *vacA* ^d^ | 346F-*vacA*-S | 5´-ATGGAAATACAACAAACACAC | 176/203bp | [76] |
|  | 346R-*vacA*-S | 5´-CCTGARACCGTTCCTACAGC |  |  |
| *vacA* ^d^ | 347F-*vacA*-M | 5´-CACAGCCACTTTCAATAACGA | 401/476bp | [76] |
|  | 347R-*vacA*-M | 5´-CGTCAAAATAATTCCAAGGG |  |  |
| *cagA* ^d^ | 48F-*cagA* | 5’-AAAGGATTGTCCCTACAAGAAGC | 330 bp | This study |
|  | 48R-*cagA* | 5’-GTAAGCGATTGCTCTTGCATC |  |  |
| *virB10* ^d^ | 46F-*virB10* | 5’-TGCCTTTGGAAGATAAAAGCTC | 350 bp | This study |
|  | 46R-*virB10* | 5’-TTCAGTTTTGGCATTTTTGATG |  |  |
| *virB11* ^d^ | 10F-*virB11* | 5’-CCCTGTTACAGTTAATGATGAAACC | 347 bp | This study |
|  | 10R-*virB11* | 5’-ACTTTAAGCAATCAGCAGAGGTG |  |  |
| RAPD | D-8635 | 5´-GAGCGGCCAAAGGGAGCAGAC | - | [27] |
|  | D-9355 | 5´-CCGGATCCGTGATGCGGTGCG | - | [27] |
|  | D-14307 | 5´-GGTTGGGTGAGAATTGCACG | - | [27] |
|  | 1254 | 5´-CCGCAGCCAA | - | [33] |
|  | 1281 | 5´-AACGCGCAAC | - | [33] |
|  | 1283 | 5´-GCGATCCCCA | - | [33] |
|  | 1290 | 5´-GTGGATGCGA | - | [33] |

# ^a^ For mixed bases, the following code was used:

# A/G=R, A/C=M, A/T =W, G/O= S, G/T=K, C/T=Y, A/G/O=V, A/G/T=D, A/C/T=H, G/C/T=B

^b^ Primers based on *Helicobacter* genus sequences [53].

^c^ Primers based on *Helicobacter acinonychis* Sheeba sequences [31].

^d^ Primers based on *Helicobacter pylori* 26695 sequences [28].

^e^ Reference [90], not listed in main text: Dewhirst FE, Shen Z, Scimeca M, [Stokes LN](http://www.ncbi.nlm.nih.gov/pubmed?term=Stokes LN%5BAuthor%5D&cauthor=true&cauthor_uid=16109952), [Boumenna T](http://www.ncbi.nlm.nih.gov/pubmed?term=Boumenna T%5BAuthor%5D&cauthor=true&cauthor_uid=16109952), et al. (2005) Discordant

16S and 23S rRNA phylogenies for the genus *Helicobacter*: implications for phylogenetic inference and systematics. J Bacteriol 187:

6106–6118.
